# Single-cell transcriptomic classification of rabies-infected cortical neurons

**DOI:** 10.1073/pnas.2203677119

**Published:** 2022-05-24

**Authors:** Maribel Patiño, Will N. Lagos, Neelakshi S. Patne, Bosiljka Tasic, Hongkui Zeng, Edward M. Callaway

**Affiliations:** ^a^Systems Neurobiology Laboratories, The Salk Institute for Biological Studies, La Jolla, CA 92037;; ^b^Neuroscience Graduate Program, University of California San Diego, La Jolla, CA 92093;; ^c^Medical Scientist Training Program, University of California San Diego, La Jolla, CA 92093;; ^d^Allen Institute for Brain Science, Seattle, WA 98109

**Keywords:** rabies virus, transcriptomics, cell types, circuit tracing, gene expression

## Abstract

Cortical circuit tracing using modified rabies virus can identify input neurons making direct monosynaptic connections onto neurons of interest. However, challenges remain in our ability to establish the cell type identity of rabies-labeled input neurons. While transcriptomics may offer an avenue to characterize inputs, the extent of rabies-induced transcriptional changes in distinct neuronal cell types remains unclear, and whether these changes preclude characterization of rabies-infected neurons according to established transcriptomic cell types is unknown. We used single-nucleus RNA sequencing to survey the gene expression profiles of rabies-infected neurons and assessed their correspondence with established transcriptomic cell types. We demonstrated that when using transcriptome-wide RNA profiles, rabies-infected cortical neurons can be transcriptomically characterized despite global and cell-type-specific rabies-induced transcriptional changes. Notably, we found differential modulation of neuronal marker gene expression, suggesting that caution should be taken when attempting to characterize rabies-infected cells with single genes or small gene sets.

## Significance

Monosynaptic rabies tracing using glycoprotein (G)-deleted rabies virus is widely applied to study cortical circuit connectivity. However, connectivity tracing using rabies virus could benefit from higher throughput methods of assigning rabies-labeled inputs to neuronal cell types and the ability to assign cells to finer genetically defined subtypes. Using single-nucleus RNA sequencing, we demonstrate that rabies-infected cortical neurons can be transcriptomically characterized according to established cell types when using transcriptome-wide analysis. Furthermore, we find that rabies infection differentially affects distinct host genes, suggesting that some genes may be more vulnerable to transcriptional modulation than others, which may impede the classification of rabies-infected cells when using methods that rely on the detection of single or few genes.

Monosynaptic rabies tracing using glycoprotein (G)-deleted rabies virus has been widely adopted for circuit tracing studies throughout the central and peripheral nervous systems and has had a great impact on the understanding of neural circuit organization (1, 2). This approach allows for the identification of the direct presynaptic inputs to specific cell types (3) or to single neurons of interest (4) across the whole brain. Recent advances in single-cell transcriptomics have profoundly altered our views of the numbers and diversity of genetically defined cell types (5), raising important questions about circuit organization at the level of these cell types and introducing new challenges for determining the identity of retrogradely labeled input cells. While some studies have successfully used rabies tracing to identify the cell-type-specific inputs to specific types of cortical neurons (6, 7), classification of inputs relied either on antibody staining or intersectional methods using Cre-Flp intersectional fluorescent reporters. The former approach drastically limits the cell types that can be characterized because their identification relies on antibody availability and specificity and may also be subject to artifacts from the translation-inhibiting properties of the rabies M protein (8). The latter methods, while not subject to similar artifacts, are cumbersome, allowing for the interrogation of only one cellular input type per experimental animal and requiring multiple experiments and Cre mouse lines to determine the identity of all input cells. Overall, connectivity tracing using rabies virus could benefit from higher throughput methods of assigning inputs to neuronal cell types and the ability to assign cells to finer subtypes.

One way to achieve these goals is to combine single-cell transcriptomics with monosynaptic rabies circuit tracing. Single-cell RNA sequencing (scRNA-seq) technologies have begun to expose the full extent of cortical cell type diversity and findings suggest that previously defined cell types may be composed of multiple distinct types. Studies utilizing scRNA-seq to study neuronal cell type diversity across cortical areas have found large numbers of clusters corresponding to putative cell types (9, 10). In one study (11), scRNA-seq data from more than 23,000 neurons from the primary visual cortex (V1) and the anterior lateral motor cortex revealed 133 distinct transcriptomic cell types, as follows: 61 inhibitory, 56 glutamatergic, and 16 nonneuronal types. Transcriptomic analyses revealed that each of the major cortical neuronal inhibitory classes could be further divided into multiple inhibitory cell subtypes, including 20 somatostatin (Sst), 10 parvalbumin (Pvalb), 16 vasoactive intestinal peptide (Vip), and 13 ionotropic serotonin receptor (HTR3A)+/VIP− clusters (belonging to Lamp5 and Sncg subclasses). Furthermore, multiple studies are beginning to establish the transcriptomic correlates of functional, electrophysiological, and morphological diversity within and across excitatory and inhibitory neurons (12–14). Together, these studies demonstrate that this transcriptomic diversity is meaningful and that the transcriptomic characterization of neurons can be beneficial in untangling the precise circuit connections underlying cortical function.

Monosynaptic rabies tracing combined with single-cell transcriptomics could be a more efficient and precise method for determining the cell type identity of inputs to populations of interest than current methods. However, rabies virus infection is known to cause changes in host cellular transcription. Studies show that a major effect of rabies infection in the mouse brain is the global down-regulation of gene expression and up-regulation of genes involved in immune responses (15–17). However, these studies were either performed on bulk tissue and could not investigate the differential effects of infection across different cell types or they focused their scRNA-seq analysis on non-neuronal cells at the infection site rather than on confirmed rabies-infected neurons. Therefore, the extent of transcriptional changes induced in distinct neuronal cell types and whether these changes preclude characterization of rabies-infected neurons according to established transcriptomic cell types remain unknown. Here, we used single-nucleus RNA sequencing (snRNA-seq) to assess the correspondence between rabies-infected and uninfected neuronal nuclei and investigated the effects of rabies infection on neuronal marker genes. We found that, despite global and cell-type-specific rabies-induced transcription changes, both neuronal and nonneuronal rabies-infected cells can still be classified according to established transcriptomic cell types. Furthermore, we show that rabies differentially affects the expression of neuronal marker genes, with certain marker genes being up-regulated or down-regulated and others remaining unperturbed. Of note, the canonical cortical inhibitory interneuron marker genes *Sst*, *Pvalb*, and *Vip* were strongly down-regulated in rabies-infected nuclei. Our findings suggest that rabies tracing is compatible with transcriptomic characterization of input cells when utilizing transcriptome-wide RNA profiles, such as those obtained with snRNA-seq. We further illustrate that caution should be taken when attempting to characterize rabies-labeled cells with single marker genes or proteins. Finally, we have made our dataset of rabies-infected nuclei publicly available to serve as a resource to determine which genes may be most suitable for establishing rabies-labeled input identities with methods that rely on single genes or discrete gene sets, such as spatial transcriptomics or RNA fluorescence in situ hybridization (FISH).

## Results

### Rabies Infection Induces Global Transcriptional Changes in Mouse V1.

**Fig. 1.**
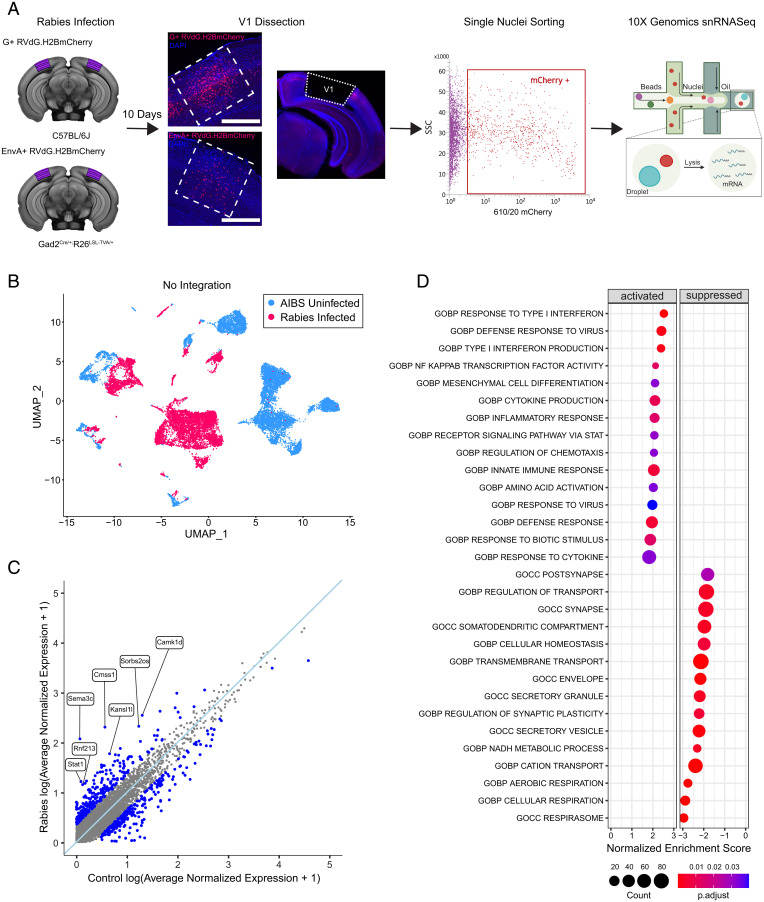
Rabies infection induces global transcriptional changes in mouse V1. (*A*) Schematic of experiment workflow. C57BL/6 mice (*n* = 3, 2 females and 1 male) and Gad2Cre/+; R26LSL-TVA/+ (*n* = 8, 5 females and 3 males) were injected with unpseudotyped G+RVdG. H2B.mCherry and pseudotyped EnvA+RVdG.H2BmCherry, respectively. At 10 days after injection, V1 was dissected and mCherry+ nuclei collected using FANS. snRNA-seq of FANS–sorted rabies-infected nuclei was performed using the 10× Genomics platform. Scale bar: 500 µM. (*B*) UMAP of 8,745 rabies-infected nuclei (red) and 9,508 AIBS uninfected control nuclei (blue) merged without integration analysis. (*C*) Scatter plot displaying gene expression differences in rabies-infected nuclei versus AIBS uninfected control. The light-blue line indicates a perfect correlation. Genes with log_2_FC of >0.25 are highlighted in blue, and genes most up-regulated in the rabies-infected group are labeled. Values were averaged log normalized across all cells in each condition. (*D*) Dot plot of the top GO gene sets significantly (padj < 0.05, Benjamini–Hochberg correction) activated (*Left*) or suppressed (*Right*) in the rabies-infected dataset compared with AIBS uninfected control.

To investigate the transcriptional response to rabies infection, we compared the transcriptomes of rabies-infected nuclei from mouse V1 with those from uninfected controls. Rabies-infected nuclei were collected from V1 of wild-type C57BL/6 mice (*n* = 3, 2 females and 1 male) injected with unpseudotyped SAD-B19 G-deleted rabies virus expressing nuclear-localized mCherry (G+RVdG.H2B.mCherry) at postnatal day 60 (P60) to P75. Because excitatory neurons comprise about 80% of all cortical neurons (18), the majority of nuclei obtained with this approach were from excitatory neurons. To enrich for rabies-infected inhibitory neurons, Gad2-Cre mice were crossed to R26-LSL-TVA-LacZ mice (19) to express the avian tumor virus receptor A (TVA), which is necessary for viral entry of EnvA-pseudotyped rabies virus, in inhibitory neurons. These transgenic mice (*n* = 8, 5 females and 3 males) were then injected with EnvA-pseudotyped G-deleted rabies virus expressing nuclear-localized mCherry (EnvA+RVdG.H2BmCherry) at P65 to P85, which resulted in direct and selective rabies infection of the targeted inhibitory cell class (Fig. 1*A*). The biased samples allowed more complete coverage of the rarer but more diverse inhibitory neuron types. At 10 days after injection, V1 containing mCherry+ rabies-labeled nuclei was dissected and single-nucleus suspensions were prepared from dissected tissue for fluorescence-activated nuclei sorting (FANS) to collect mCherry+ nuclei (Fig. 1*A* and *SI Appendix*, Fig. S1 *A* and *B*). snRNA-seq of FANS-sorted rabies-infected nuclei was performed using the 10× Genomics 3′ Kit v3.1. The rabies dataset was compared with an independent snRNA-seq dataset composed of uninfected nuclei collected from V1 of control uninjected mice acquired using the 10× Genomics 3′ Kit v3. The uninfected control dataset contained cell type annotations established according to the Allen Institute for Brain Science (AIBS) cell type taxonomy (11). Following quality-control filtering, principal-component analysis (PCA), and unsupervised graph-based clustering of 8,745 rabies-infected and 9,508 uninfected control nuclei, we applied Uniform Manifold Approximation and Projection (UMAP) to visualize gene expression relationships across infection status. The resulting UMAP plot illustrates that nuclei were grouped primarily by their infection status, with infected and uninfected neurons forming clearly separated clusters (Fig. 1*B*). To examine the global response to rabies infection and identify possible gene expression differences between rabies-infected and -uninfected nuclei, we performed differential expression (DE) analysis using Model-based Analysis of Single-Cell Transcriptomics (MAST) (20). Up-regulated differentially expressed genes (DEGs; adjusted *P* < 0.05; log_2_ fold change [FC] > 0.25) (Fig. 1*C* and *SI Appendix*, Table S1) included interferon response genes, such as *Stat1* and *Stat2*, involved in JAK-STAT signaling pathways, a pathway implicated in antiviral and innate immune responses. To better understand the possible functions of up-regulated and down-regulated genes in rabies-infected nuclei, we performed gene set enrichment analysis (GSEA). GSEA using Gene Ontology (GO) revealed that up-regulated DEGs were highly enriched in viral response, innate immunity, and inflammatory pathways such as type 1 interferon signaling, cytokine production and response, and nuclear factor kappa B (NF-κB) signaling (Fig. 1*D*). Down-regulated genes were predominantly involved with cellular respiration, metabolic pathways, and cellular homeostasis. Additionally, down-regulated genes were also enriched for genes encoding proteins involved in synaptic and somatodendritic compartments. These findings are consistent with prior bulk and scRNA-seq studies exploring global or nonneuronal transcriptional changes following rabies infections (15, 16, 21).

### Rabies-Infected Nuclei Can Be Transcriptomically Classified Despite Changes in Gene Expression.

**Fig. 2.**
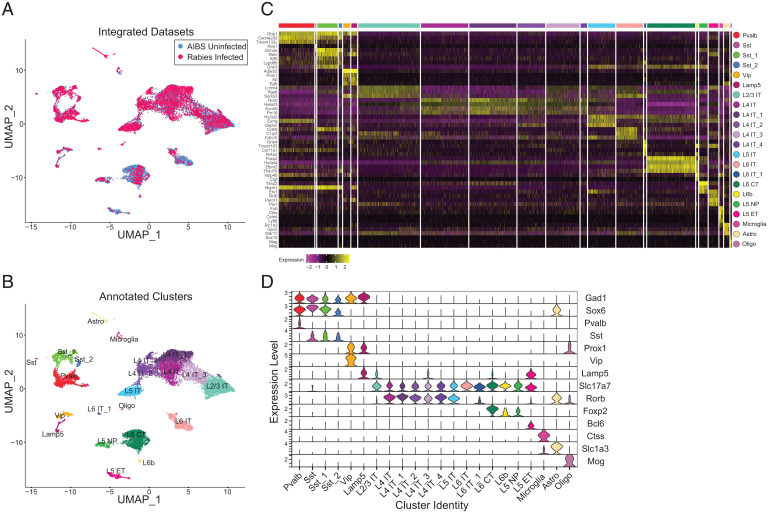
Rabies-infected nuclei can be transcriptomically classified de novo despite changes in gene expression. (*A*) UMAP of 8,745 rabies-infected nuclei (red) and 9,508 AIBS uninfected control nuclei (blue) after anchor-based data integration. (*B*) UMAP of anchor-based integrated rabies-infected and AIBS uninfected control nuclei colored by de novo cell subclass annotations. (*C*) Heatmap showing normalized and scaled expression level of DEGs for each cluster in *B* compared with all other clusters. Clusters are color coded according to *B* and delineated by white vertical lines. (*D*) Violin plots illustrating normalized expression level of canonical marker genes for each cluster, which are composed of both infected and AIBS uninfected control nuclei.

To characterize rabies-infected nuclei according to established transcriptomic cortical cell types, we used computational strategies for an integrated analysis of snRNA-seq datasets that have been shown to enable comparisons of heterogeneous tissue across different experimental conditions (22, 23). Anchor-based data integration was used to identify anchors representing cells in similar biological states across datasets, which were used to guide the merging of the rabies-infected and uninfected control datasets. UMAP visualization of the integrated datasets confirmed that nuclei cluster according to biological cortical cell types and not by infectious status (Fig. 2*A*). Clustering analysis segregated nuclei into 22 clusters (Fig. 2*B*). Based on known marker genes for major neuronal and nonneuronal classes, we identified 6 inhibitory neuron clusters that express GABAergic inhibitory interneuron markers *Gad1* and *Gad2*; 13 excitatory neuron clusters that express *Slc17a7*, which encodes the vesicular glutamate transporter VGLUT1; and 3 nonneuronal clusters corresponding to microglia, astrocytes, and oligodendrocytes expressing *Ctss*, *Slc1a3*, and *Mog*, respectively. The proportion of excitatory (uninfected = 85.57% vs. rabies = 75.38%), inhibitory (uninfected = 13.66% vs. rabies = 20.86%), and nonneuronal nuclei (uninfected = 0.76% vs. rabies = 3.76%) was similar across experimental groups (*SI Appendix*, Fig. S2*A*) with a slightly higher proportion of inhibitory neurons in the rabies dataset, likely resulting from interneuron enrichment. We used DEGs (Fig. 2*C*) and previously reported cell type markers (Fig. 2*B*) to further annotate clusters into cell subclasses. We identified four inhibitory neuronal clusters derived from the medial ganglionic eminence (MGE), expressing *Lhx6*/*Sox6*, and two clusters derived from the caudal ganglionic eminence (CGE), expressing *Adarb2*/*Prox1*. MGE-derived clusters included one Pvalb and three Sst clusters. CGE-derived clusters included one Vip and one *Lamp5^+^* neurogliaform cluster. For glutamatergic neurons, we identified major subclasses previously reported (11), including L2/3, L4, L5, and L6 intratelencephalically (IT)-projecting clusters, expressing a combination of markers including *Cux2*, *Fam19a1*, *Rorb*, and *Deptor*. Additional glutamatergic clusters included L5 near projecting (NP), L5 extratelencephalically (ET) projecting, L6b, and L6 corticothalamic (CT). The distribution of rabies-infected and uninfected nuclei in each cluster (*SI Appendix*, Fig. S2*B*) show a larger proportion of rabies-infected nuclei than the control in MGE-derived clusters, possibly as a result of interneuron enrichment using Gad2-Cre; R26-LSL-TVA mice that would bias rabies infection to inhibitory neurons, 40% of which are Pvalb and 18% of which are Sst (24). Additionally, there was an enrichment of L4 and L5 rabies-infected neurons that may be the result of infection biases arising from injecting rabies at a cortical depth of 0.5 mm (Fig. 1*A*).

**Fig. 3.**
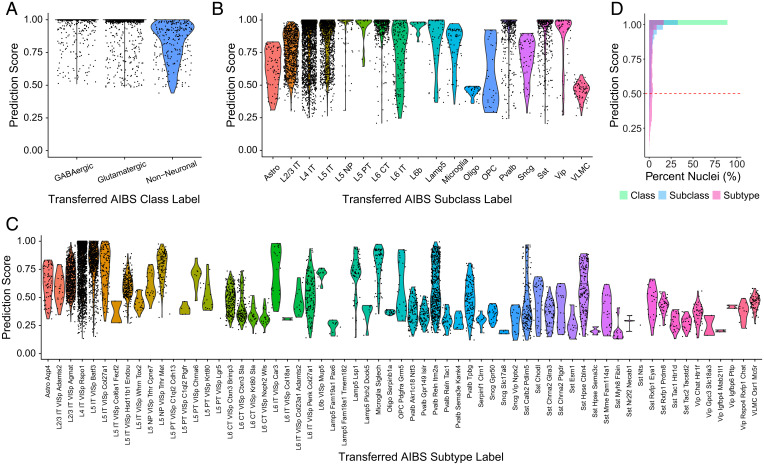
Preexisting cell type annotations can be transferred to rabies-infected nuclei. (*A*) Violin plots of prediction classification scores obtained when transferring AIBS class labels to rabies-infected nuclei. (*B*) Violin plots of prediction classification scores obtained when transferring AIBS subclass labels to rabies-infected nuclei. (*C*) Violin plots of prediction classification scores obtained when transferring AIBS subtype labels to rabies-infected nuclei. (*D*) Distribution of cell type prediction scores, ranging from 0 to 1, for rabies-infected nuclei at different granularities. Dashed line indicates high-confidence predictions (score of >0.5).

In addition to transcriptomically classifying rabies-infected nuclei de novo using unsupervised graph-based clustering, we also examined whether the AIBS taxonomy cell type labels could be transferred from the reference snRNA-seq dataset of uninfected cells to the rabies-infected nuclei using a weighted vote classifier derived from the reference cell identities. This approach provides a quantitative score, ranging from 0 to 1, for each predicted cell type classification, with high-confidence cell type predictions being those greater than 0.5 (23). We tested the transfer of reference class (Fig. 3*A*), subclass (Fig. 3*B*), and subtype (Fig. 3*C*) labels. At the class level, 100% of rabies-infected nuclei were classified with a prediction score of >0.5 (Fig. 3*D*). The ability to confidently assign cell type predictions decreased at finer cell type granularities but overall was successful, with 95% and 75% of rabies-infected nuclei having a prediction score of >0.5 at the subclass and subtype level (Fig. 3*D*). At the subtype level, the distributions of prediction scores varied considerably between subtypes (Fig. 3*C*). This variability likely results from variability in the sample size for each subtype in the reference, with some subtypes being underrepresented in the reference. Thus, it may be possible to improve the subtype assignment of rabies-infected cells provided that there is a large enough reference sample.

### Cell-Type–Specific Rabies-Induced Transcriptional Changes.

**Fig. 4.**
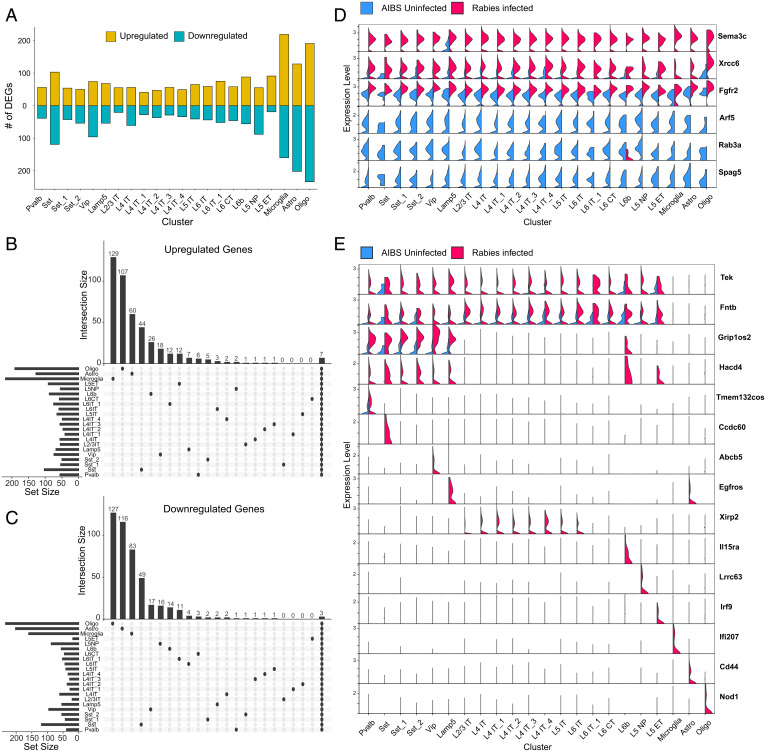
Cell-type–specific rabies-induced transcriptional changes. (*A*) Bar plot showing the number of DEGs that are up-regulated (yellow) or down-regulated (blue) in each cluster in rabies-infected nuclei compared with AIBS control. (*B* and *C*) Upset plots of up-regulated (*B*) and down-regulated (*C*) DEGs in rabies infected versus AIBS control. Horizontal bars represent the number of DEGs detected in each cluster, and vertical bars represent the number of unique DEGs in that cluster or in selected intersections between clusters indicated below the bars. (*D*) Violin plots displaying normalized expression of select DEGs in rabies-infected nuclei and AIBS control nuclei in each cluster. Top three genes are up-regulated in rabies-infected nuclei across all clusters and bottom three genes are down-regulated in rabies-infected nuclei across all clusters. (*E*) Violin plots displaying normalized expression of select DEGs in rabies-infected nuclei and AIBS control nuclei in each cluster. Genes are differentially expressed in neuronal clusters (top two rows), mainly inhibitory clusters (third row), or in unique clusters.

To explore whether rabies infection induces cell-type–specific transcriptional changes, we performed DE analysis separately for each of the identified clusters. We observed DEGs in each cluster (adjusted *P* < 0.05; log_2_FC > 1), with nonneuronal cells containing a higher number of DEGs compared with neuronal cells (Fig. 4*A* and *SI Appendix*, Table S2). Of these DEGs, only 7 and 3 were up-regulated and down-regulated, respectively, across all clusters (Fig. 4 *B* and *C*). The overwhelming majority of DEGs were differentially expressed only in subsets of clusters or single clusters. Genes that shared expression up-regulation across all clusters included genes involved in antiviral and immune responses, such as *Xrcc6* that acts as a cytosolic viral sensor and mediates downstream immune responses (25, 26) and *Fgfr2* that regulates RIG-1-mediated antiviral signaling (27) (Fig. 4*D*). We also observed a global up-regulation of *Sema3c*, which encodes Semaphorin 3C (Sema3c), a soluble axonal chemoattractant (28). *Sema3c* has been reported to be selectively expressed in neurogliaform inhibitory interneurons (29) and serves as a neuronal marker for this subclass (30). Indeed, in control nuclei, only the *Lamp5^+^* neurogliaform cluster shows expression of *Sema3c*; however, selective expression is lost and up-regulation is observed in all clusters for rabies-infected nuclei (Fig. 4*D*). Genes down-regulated in most clusters included those involved in Golgi vesicle transport, such as *Arf5,* and cellular transmembrane transport, such as *Rab3a* and *Spag5*. Although some DEGs were part of a global response across all clusters, other genes were differentially expressed only in specific clusters or classes (Fig. 4*E*). For example, *Tek*, *Fntb*, and *Itgl1* were selectively up-regulated in neuronal clusters. Similarly, *Grip1os2* was selectively up-regulated in inhibitory and L6b neurons and *Xirp2* in IT excitatory clusters.

### Effects of Rabies Infection on Neuronal Marker Genes.

**Fig. 5.**
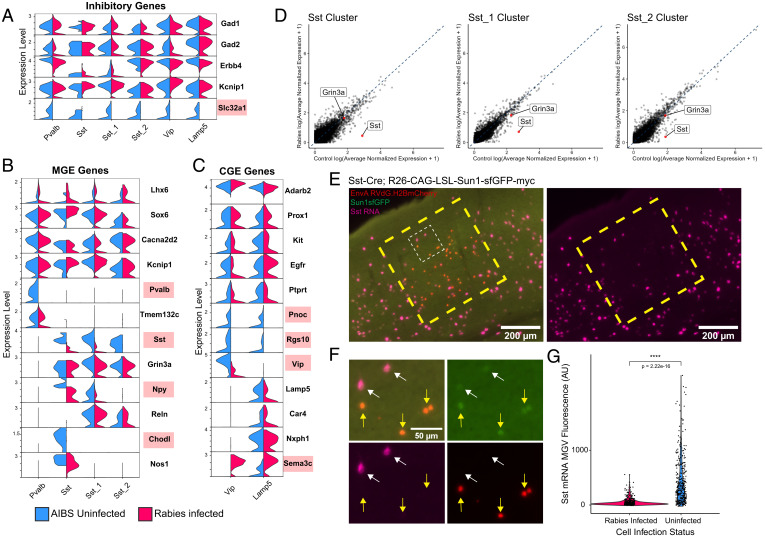
Rabies infection differentially affects the expression of inhibitory marker genes. (*A*–*C*) Violin plots displaying normalized expression of select inhibitory (*A*), MGE (*B*), or CGE (*C*), marker genes in rabies-infected nuclei and AIBS control nuclei in each cluster. Marker genes up-regulated or down-regulated in rabies-infected compared with AIBS control are highlighted in red. (*D*) Scatter plot displaying expression differences in rabies-infected nuclei versus AIBS control in Sst clusters, with Sst and Grin3a labeled in red. The dashed blue line indicates perfect correlation. Values were averaged log normalized across cells in each condition in that cluster. (*E*) Representative images of HCR labeling for Sst transcripts (magenta) in V1 of Sst Cre;R26-CAG-LSL-Sun1-sfGFP-myc mice injected with EnvA RVdG.H2BmCherry. Uninfected Sst neurons are Sun1sfGFP+, mCherry−, and rabies-infected Sst neurons are Sun1sfGFP+, mCherry+. Large dashed yellow box indicates a region containing rabies-infected Sst neurons. The small dashed white box indicates the region shown in *F* with higher magnification. Scale bar: 200 µm. (*F*) Higher magnification of dashed white box region in *E*. White arrows indicate uninfected Sst neurons (Sun1sfGFP+, mCherry−,) and yellow arrows indicate rabies-infected Sst neurons (Sun1sfGFP+, mCherry+). Sst transcripts labeled with HCR are in magenta. Scale bar: 50 µm. (*G*) Violin plots displaying MGV intensity of Sst transcript fluorescence. A total of 356 rabies-infected and 347 uninfected Sst neurons across 2 animals were analyzed. *P* values were determined by Wilcoxon rank-sum test. ****P ≤ 0.0001.

We next focused the analysis on examining rabies effects on genes commonly used to define neuronal cell types. The expression of select marker genes, such as *Gad1* and *Gad2*, used to distinguish inhibitory neurons from excitatory neurons and nonneuronal cells was unperturbed by rabies infection. However, *Slc32a1*, expressed selectively in interneuron clusters, was down-regulated in rabies-infected nuclei (Fig. 5*A*). MGE marker genes *Lhx6*, *Sox6*, *Cacna2d2*, and *Kcnip1* were not differentially expressed (Fig. 5*B*). Similarly, some marker genes of CGE neurons were unaffected such as *Adarb2*, *Prox1*, and *Kit*, whereas *Pnoc* and *Rgs10* were down-regulated (Fig. 5*C*). Importantly, *Pvalb* and *Sst*, crucial marker genes of inhibitory interneurons, were strongly down-regulated in rabies-infected nuclei (Fig. 5 *B* and *D*). However, other marker genes differentially expressed between Pvalb and Sst subclasses remained unchanged, such as *Tmem132c* and *Grin3a*, respectively. Similar to class and subclass markers, rabies differentially affected markers of neuronal subtypes. The Sst-Chodl neuron subtype corresponds to long-range projecting inhibitory neurons commonly found in cortical layer 5 and 6 that express high levels of nitric oxide synthase (*Nos1*) (11, 30). Within this cluster, rabies down-regulated the expression of the unique marker *Chodl* but did not affect the expression of *Nos1*. The expression of *Vip* was also down-regulated, but *Lamp5*, *Car4*, and *Nxph4* were unaffected. Interestingly, neurogliaform marker gene *Sema3c* was up-regulated not only within the *Lamp5^+^* cluster but also in clusters that do not typically express it.

Although rabies infection down-regulated the expression of a variety of interneuron marker genes, *Sst* was selected for further validation with hybridization chain reaction RNA FISH (HCR RNA-FISH). Prior studies characterizing rabies-labeled input cells have found conflicting results regarding the ability of rabies virus to infect Sst interneurons (6, 7). Therefore, *Sst* was selected for in vivo validation to attempt to address this inconsistency and investigate whether rabies-induced *Sst* expression down-regulation may be an artifact leading to detection failure. To allow for the identification of Sst interneurons after rabies infection without relying on methods possibly subject to expression artifacts, Sst-Cre mice were first crossed to R26R-CAG-loxp-stop-loxp-Sun1-sfGFP-Myc (31) mice to produce Sst-Cre; INTACT, resulting in nuclear super folder GFP (sfGFP) expression in Sst interneurons. These mice were injected with adeno-associated virus (AAV) to express Cre-dependent TVA followed by EnvA+RVdG-H2BmCherry to restrict rabies infection to Sst interneurons. Importantly, this experimental design makes it possible to quantify and compare *Sst* RNA in uninfected and rabies-infected Sst interneurons in the same mouse and tissue section (Fig. 5 *E* and *F*). In agreement with our snRNA-seq data, we found that *Sst* RNA detection by HCR RNA-FISH is significantly reduced in rabies-infected versus -uninfected Sst interneurons (Fig. 5*G*).

**Fig. 6.**
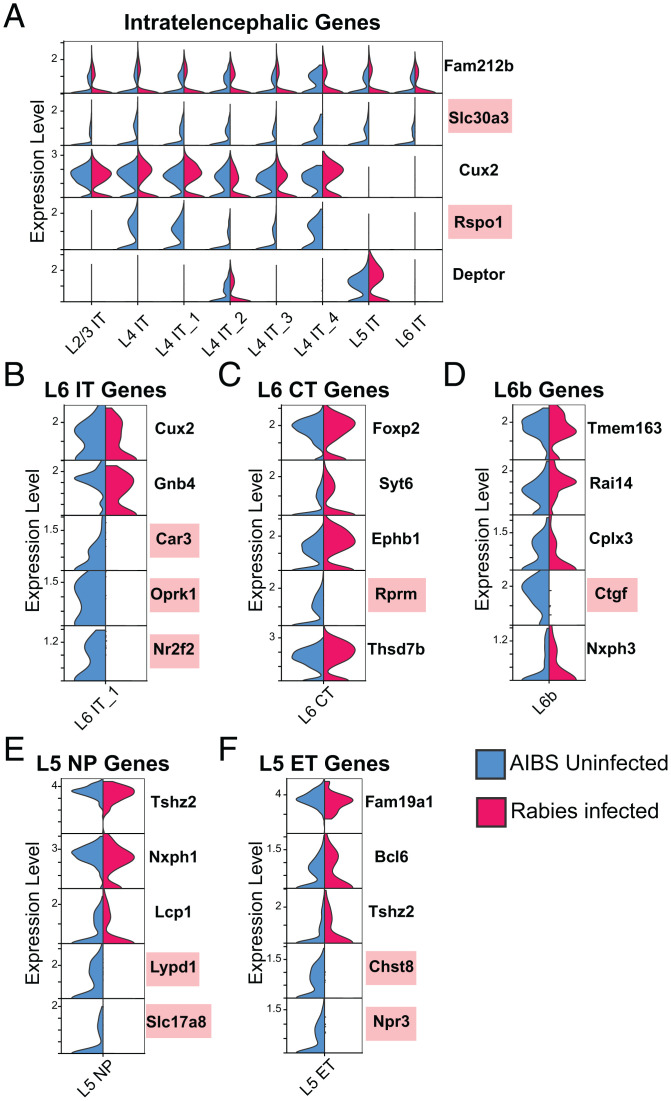
Rabies infection differentially affects the expression of excitatory marker genes. (*A–F*) Violin plots displaying normalized expression of select excitatory IT (*A*), L6 IT (*B*), L6 CT (*C*), L6b (*D*), L5 NP (*E*), and L5 ET (*F*) marker genes in rabies-infected nuclei and AIBS control nuclei in each cluster. Down-regulation of select marker genes (red) is observed in rabies-infected nuclei.

Similar to its effects on inhibitory marker genes, rabies infection differentially affected excitatory neuronal markers (Fig. 6). Important excitatory marker genes down-regulated in rabies-infected nuclei included *Slc30a3* and *Rspo1*, which are marker genes for pan-IT neurons and L4 IT neurons, respectively (Fig. 6*A*). Layer-specific IT marker genes not altered included *Cux2*, a L2/3 IT marker gene, and *Deptor*, a L5 IT maker gene. The L6 IT Car3 subclass is transcriptomically distinct from other excitatory IT subclasses (11), and Car3+ L6 cells have been shown to exhibit distinct projection patterns compared with other IT-projecting neurons (32). We found that previously reported marker genes for this subclass, namely, *Car3*, *Oprk1*, and *Nr2f2*, are all down-regulated in rabies-infected nuclei within this cluster (Fig. 6*B*). However, *Gnb4*, a marker also unique for the Car3 subclass, and *Cux2*, a gene expressed in L6-IT-Car3, but absent in other L6-IT neurons, remain unchanged. Within the L6 CT subclass, canonical marker gene *Foxp2* was not affected by rabies expression along with *Thsd7b*, *Syt6*, and *Ephb* (Fig. 6*C*). L6b neurons share marker genes such as, *Cplx3*, *Nxph3*, and *Ctgf*, of which the latter is down-regulated in rabies-infected nuclei. L5 NP neurons project only to neighboring areas and express distinct markers such as *Slc17a8*, *Tshz2*, and *Lypd1.* Like other clusters, rabies down-regulated certain genes (*Slc17a8*, Lypd1) while having no effect on others (*Tshz2*, *Lcp1*). Within the L5 ET subclass, we found down-regulation of *Npr3* and *Chst8* and no change in the expression of *Bcl6*, *Fam19a1*, and *Tshz2*. Overall, we found that rabies infection can alter the expression of certain neuronal marker genes, while others are unchanged. Furthermore, when gene expression is altered, the most common effect observed is gene expression down-regulation, although up-regulation of some marker genes was also present.

## Discussion

Monosynaptic rabies tracing is a valuable tool for unraveling neural connectivity, and yet challenges remain in the ability to characterize rabies-labeled input cells to populations of interest. Here, we investigated whether single-cell transcriptomics could be used to characterize rabies-infected cells according to established cell types and examined how rabies infection alters host cells’ transcriptional landscape. Using snRNA-seq to examine the transcriptomes of large numbers of rabies-infected neurons, we found that despite global and cell-type–specific rabies-induced transcriptional changes, rabies-infected neuronal profiles retain sufficient similarities to control profiles to allow for their classification. Furthermore, we examined the effects of rabies infection on neuronal marker genes and found that rabies differentially impacts host genes, with some showing down-regulation or up-regulation of expression and others remaining unperturbed. Therefore, while our study supports the use of rabies tracing in combination with snRNA-seq to determine input cell identity, it also suggests that caution should be taken when attempting to determine rabies-labeled cell identity with methods that rely on single or few genes. Finally, our dataset can serve as a resource for other researchers to utilize while designing gene probes for spatial transcriptomics on rabies-infected cortical tissue.

The wide adoption of genetically modified rabies virus to study neural connectivity can be attributed to its ability to overcome the challenge of precisely labeling presynaptic partners regardless of distance, with EnvA-pseudotyped rabies virus having the additional advantage of targeting initial infection to a genetically accessible population of interest. However, circuit connectivity analysis using this viral tool is largely restricted to characterizing inputs according to the cortical area they reside in and/or their laminar spatial location (33–35). While some studies have used immunostaining or intersectional approaches to determine the identity of rabies-labeled cortical inputs to populations of interest, studies have yielded conflicting results. For example, a study using rabies tracing to detect inputs to interneurons in mouse V1 used Sst RNA probes and antibodies to establish the identity of rabies-labeled inputs (6). Using this method, they found no Sst-positive inputs to Pvalb interneurons, despite functional studies describing extensive Sst innervation of Pvalb neurons (29). A plausible explanation is that Sst interneurons are resistant to retrograde infection by rabies virus. However, this seems unlikely given that rabies-labeled Sst inputs can be detected using a Cre/Flp recombinase-dependent intersectional labeling method where mice with a Cre/Flp-dependent dual RFP reporter and a Cre gene inserted into an interneuron subtype-specific gene locus are used in tandem with pseudotyped rabies viruses expressing Flp (7). Given our finding that rabies infection can down-regulate *Sst* gene expression, along with reports that rabies can also decrease the translation of host proteins, it is likely that the inability to detect Sst inputs is an artifact of using immunostaining and RNA FISH for a gene that is down-regulated in rabies-infected cells.

Despite the strong down-regulation of certain neuronal marker genes, gene expression of many other marker genes was unaltered. This suggests that rabies infection differentially affects distinct host genes and that some genes may be more vulnerable to transcriptional modulation than others. Nonetheless, when using whole-transcriptome information and defining cell types based on expression levels of thousands of genes, the majority of rabies-infected neurons were accurately classified at the class (100%), subclass (95%), and subtype (75%) level using supervised classification to a reference data set. It is possible that the accuracy of assignment to particular subtypes may be further improved by increasing the size of the reference sample, particularly for rare cell types, as some subtypes were not well represented in the reference.

The ability to characterize rabies-infected cells based on their gene expression profiles can have large implications for future cortical connectivity studies. A multitude of studies using single-cell transcriptomics have already shown that major neuronal classes are composed of multiple genetically distinct subclasses. This has corroborated earlier studies describing distinct subtypes of interneuron classes each with unique morphology, connectivity, and functional activity (14). Thus, single-cell transcriptomics as a tool to characterize rabies-labeled inputs is not only less liable to technical artifacts but also has the additional benefit of providing a more precise way to study cortical connectivity since inputs can be identified based on more refined subtypes. Furthermore, emerging genomic technologies, such as multiplexed error-robust FISH (MERFISH), sequential FISH (seqFISH), and spatial barcoding, can transcriptomically profile many cells while retaining spatial information and may thus be valuable tools for relating molecular profiles, spatial locations, and projection patterns when used in combination with rabies tracing. However, these techniques do not yet yield deep transcriptomic information but instead rely on the expression of discrete gene sets and may therefore be more liable to artifacts induced by rabies infection. Here, we have shown that rabies differentially affects neuronal marker genes and have provided examples of genes down-regulated or up-regulated by rabies infection. Importantly, rabies-induced changes in gene expression make certain marker genes unsuitable for the accurate characterization of rabies-infected cells if used in isolation. For example, *Sst* RNA probes will not accurately label rabies infected Sst neurons due to down-regulation of the gene. Similarly, using probes against the neurogliaform marker gene *Sema3c* would likely lead to the mischaracterization of neurons, as its global up-regulation would make it detectable in nonneurogliaform neurons. Therefore, we encourage investigators to independently validate the utility of reagents that detect single genes for the characterization of rabies-infected neurons. Additionally, we have provided examples of neuronal marker genes for major classes, subclasses, and select subtypes that are unperturbed by rabies and may thus serve as suitable genes to include in gene sets to be used for spatial transcriptomics following rabies tracing. For example, while *Pvalb* may not be suitable for identifying the Pvalb interneuron subclass due to its down-regulation in rabies-infected nuclei, *Tmem132c* can serve as an alternative gene that is unaffected. Similarly, *Nos1* can be used to distinguish the *Chodl* Sst subtype as *Chodl* may not be a suitable candidate. Previous studies have demonstrated the viability of rabies-infected neurons for functional studies (2, 36). While rabies-infected neurons remain viable during the 10-d survival times that we have assessed with our transcriptomic analyses, the changes in gene expression that we have observed, including down-regulation of genes related to synaptic function and metabolic processes, reiterate the need for caution when interpreting such studies.

Here, we present and make publicly available a large snRNA-seq dataset of rabies-infected neurons consisting of 8,497 neuronal profiles. However, it is important to highlight several limitations of the study. First, prior bulk RNA-seq studies have shown that different rabies virus strains induce distinct global transcriptional changes (37). Thus, it is possible that other strains used for monosynaptic rabies tracing, such as CVS-N2c (36), may induce different cell-type–specific neuronal transcriptional changes from the ones observed using the SAD-B19 strain. Future studies should examine the compatibility of other strains not included in this study with transcriptomic characterization. Second, this study only focused on investigating the effects of rabies infection at a time point of 10 d postinfection. This time point was chosen since circuit tracing experiments express rabies at a range of 7 to 10 d and we wanted to ensure that transcriptomic characterization was still possible even at the longest expression time. However, studies are needed to investigate how rabies impacts gene expression across neuronal cell types at different infection time points and whether our observations reflect a steady-state or transient state of cells. scRNA-seq studies (10, 11) have shown that transcriptomically defined excitatory and inhibitory neuronal types are generally shared across mouse cortical areas and hippocampal formation and exhibit conserved molecular signatures. Therefore, it is likely that rabies modulation of gene expression in homologous neuronal cell types across cortical regions will not vary significantly from what we have observed in V1. However, transcriptomic cell type identification of rabies-infected cells from other brain regions would necessitate the collection and analysis of data from those brain regions of interest. Finally, while we provide evidence that rabies modulates the expression of distinct genes differently, the cellular and/or viral mechanisms that lead to these differential gene expression effects remain unknown. In summary, this study shows that rabies-infected neurons can be accurately classified according to transcriptomic cell types, identifies global and cell-type–specific rabies-induced modulation of host genes, and provides access to the transcriptomic profiles of rabies-infected cortical neurons through a publicly accessible dataset.

## Materials and Methods

### Mouse Transgenic Lines.

All experimental procedures were approved by the Salk Institute Animal Care and Use Committee. C57BL/6J mice were used as wild type. Homozygous Gad2-Cre and R26-LSL-TVA-LacZ (19) mice were bred to produce Gad2-Cre; R26-LSL-TVA-LacZ. For HCR RNA-FISH experiments, the knock-in mouse line R26R-CAG-loxp-stop-loxp-Sun1-sfGFP-Myc (INTACT) was maintained on a C57BL/6J background and bred with Sst-Cre mice to produce Sst-Cre; INTACT. Mice were housed with a 12-h light and 12-h dark cycle and had ad libitum access to food and water. Both male and female mice were used for RNA-seq experiments. Only female mice were used for HCR experiments.

### Virus Preparation.

AAV8-nef-AO-66/71-TVA950 (5.25E+13 GC/mL), EnvA+RVdG-H2BmCherry (1.09E+08 IU/mL), and G+RVdG-H2BmCherry (9.59E+09 IU/mL) were produced by the Salk GT3 Viral Core.

### Animal Surgery for Virus Injection.

Mice were initially anesthetized with 2% isoflurane and maintained at 1.5% isoflurane after placement on a stereotax (David Kopf Instruments, Model 940 series) for surgery and stereotaxic injections. A small craniotomy was made with a mounted drill over the V1 of the left hemisphere using the following coordinates: 3.4 mm posterior and 2.6 mm lateral relative to bregma. To collect large numbers of rabies-infected neurons for transcriptomic experiments, 150 nL of unpseudotyped G+RVdG-H2BmCherry (9.59E+09 IU/mL) was injected into the center of V1 of P60-P75 C57BL/6 mice 0.5 to 0.7 mm ventral from the pia using a pulled glass pipette with a tip size of 30 μm connected to a 1-mL syringe with an 18G tubing adaptor and tubing. To prevent backflow, the pipette was left in the brain for 5 min after injection. To bias infection to inhibitory neurons, 150 nL of EnvA+RVdG-H2BmCherry (1.09E+08 IU/mL) was injected into P65-P85 Gad2-Cre; R26-LSL-TVA mice. For HCR experiments 100 nL of diluted AAV8-nef-AO-66/71-TVA950 (5.25E+11 GC/mL) was injected into V1 of Sst-Cre; INTACT mice. Two weeks after AAV helper virus injection, 200 nL of EnvA+RVdG-H2BmCherry (7.43E+07 IU/mL) was injected into the same site in V1. After recovery, mice were given water with ibuprofen (30mg/kg) and housed for 10 d before tissue harvest to allow for fluorescent protein expression.

### Brain Dissection and Single Nuclei Isolation.

Ten days after rabies injection, animals were euthanized with an overdose of isoflurane. Brains were extracted and immediately submerged in ice-cold slicing solution (2.5 mM KCl, 0.5 mM CaCl_2_, 7 mM MgCl_2_, 1.25 mM NaH_2_PO_4_, 110 mM sucrose, 10 mM glucose, and 25 mM NaHCO_3_) that was bubbled with carbogen. Coronal brain slices (400 µm thick) were cut using a VF-300 Compresstome instrument (Precisionary Instruments) and submerged in ice-cold slicing solution. Subregions of V1 containing mCherry+ nuclei in brain slices were microdissected out under a fluorescent dissection microscope (Olympus SZX6), transferred to microcentrifuge tubes and immediately frozen in dry ice, and subsequently stored at −80 °C. The remaining brain slices after dissection were collected, fixed with ice-cold 4% paraformaldehyde (PFA) overnight, stained with DAPI, and scanned with a 10× objective to validate correct V1 dissection using an Olympus BX63 microscope.

Single nuclei preparations were performed following a published protocol (38) with modification. In summary, the frozen brain tissues were transferred to prechilled dounce homogenizers with 1 mL NIM buffer (0.25 M sucrose, 25 mM KCl, 5 mM MgCl_2_, 10 mM Tris⋅HCl [pH 7.4], 1 mM DTT [Sigma 646563], 10 µL of protease inhibitor [Sigma P8340], and 1.5 µL of RNasin Plus RNase inhibitor [Promega, N2611]), 0.1% Triton X-100, and 10 μM DAPI and gently homogenized on ice with ice-cold pestles 10 to 15 times. The homogenate was transferred to prechilled microcentrifuge tubes and centrifuged at 3,000 rpm for 8 min at 4 °C to pellet the nuclei. The supernatant was aspirated, and the pellet was gently resuspended in ice-cold 1 mL NIM buffer and again centrifuged at 3,000 rpm for 8 min at 4 °C. The pellet was then resuspended in 450 µL of nuclei storage buffer (0.25 M sucrose, 5 mM MgCl_2_, 10 mM Tris⋅HCl [pH7.4], 1 mM DTT, and 9 µL of protease inhibitor), and filtered through a 40-μM cell strainer. The sample was incubated with 50 µL of nuclease-free bovine serum albumin to prevent nuclei clumping.

FANS of single nuclei was performed using a BD Influx sorter with a 70 μM nozzle at a 22.5 PSI sheath pressure. DAPI+/mCherry+ rabies-infected nuclei were sorted into 1.5-mL Eppendorf tubes and immediately loaded onto the 10× Genomics Chromium Controller.

### 10× Chromium RNA-Seq.

For 10× processing, we used Chromium Next Gel Beads in Emulsion (GEM) single-cell 3′ Kit v3.1 (10× Genomics, PN-1000128). We followed the manufacturer’s instructions for single-cell capture, barcoding, reverse transcription, complementary DNA (cDNA) amplification, and library construction. We targeted a sequencing depth of 100,000 reads per cell. Libraries were sequenced on Illumina NovaSeq6000, and raw read (fastq) files were aligned to the mouse pre-mRNA reference transcriptome (mm10) using the 10× Genomics CellRanger pipeline (version 5.0). Intronic reads were included in expression quantification using the *include-introns* parameter.

### RNA-Seq Data Quality Control and Clustering.

Reference data used in this study includes 10× v3 single nucleus RNA-seq from V1 obtained from the AIBS. Reference 10× v3 nuclei were assigned to previously published VISp cell type taxonomy (11) using a nearest centroid classifier based on a set of 563 markers that were detected in both datasets (expression, >0). To estimate the robustness of mapping, classification was repeated 100 times, with each time using 80% of randomly sampled markers, and the probability for each cell to map to every reference cluster was computed. R (version 4.1.1) and Seurat (version 4.0) (22, 23) were used for snRNA-seq analysis. Doublets were identified using DoubletFinder (39) and excluded from analysis. The percentage of mitochondrial transcripts for each nucleus was calculated and added as metadata to the Seurat object using *percent.mito*. Nuclei with less than 500 genes, more than 8,000 genes, and greater than 0.5% of mitochondrial genes were excluded from the analysis. After prefiltering, AIBS and rabies datasets were normalized and scaled separately using the *SCTransform* function. Variable features were first identified in each dataset individually with *SCTransform* after removing sex-specific genes, immediate early genes, and predicted gene models (gene names that start with Gm or end with Rik). The top 5,000 variable features identified independently across datasets were determined using *SelectIntegrationFeatures* and used as the input for downstream joint clustering analysis. Datasets were merged and integration anchors identified using *FindIntegrationAnchors*. These anchors were used to integrate the two datasets using *IntegrateData*. Dimensionality reduction via PCA was performed on the integrated data using *RunPCA*. The top 50 principal components were used as the input for clustering analysis using *FindClusters* with a resolution of 0.5. For unsupervised annotations, clusters were assigned cell type annotations based on the expression of a combination of known marker genes for major cell subclasses and DEGs. For the latter, MAST (20) was used to perform the DE analysis by comparing nuclei in each cluster with the rest of the nuclear profiles, with DEGs being those with Bonferroni correction (p_adj_ < 0.05; log_2_FC > 0.25). For supervised cell type annotation, we used a weighted vote classifier derived from the reference cell identities to map rabies-infected nuclei to established neuronal cell types. The rabies dataset and the AIBS dataset were integrated using the *FindTransferAnchors* function with the AIBS dataset assigned as the reference and the rabies dataset as the query. Following integration, class, subclass, and subtype labels were transferred to the rabies dataset using the *TransferData* function, providing a prediction score ranging from 0 to 1 for each class, subclass, and subtype.

### DE Analysis and GSEA.

To identify genes differentially expressed in rabies-infected nuclei compared with control, a DE analysis was performed using MAST. *P* values were adjusted using Bonferroni correction and filtered at p_adj_ < 0.05. GSEA was performed using the *GSEA* function in the R package ClusterProfiler version 4.2 (40). DEGs were ranked according to their log fold change (rabies vs. control), and the ranked list was used as input to the *GSEA* function. GO sets and pathways were obtained using the Molecular Signatures Database (MSigDB) (41, 42). p_adj_ < 0.05 (Benjamini–Hochberg correction) was considered significant.

### In Vivo Validation of Gene Expression Using HCR RNA-FISH.

Animals were perfused transcardially using phosphate-buffered saline (PBS) followed by 4% PFA. Brains were dissected out from skulls and postfixed overnight with 2% PFA and 15% sucrose in PBS at 4 °C and then immersed in 30% sucrose in PBS at 4 °C for an additional 24 h. HCR reagents and Sst probes were obtained from Molecular Instruments. Tissue was sectioned coronally at 50 µm on a freezing microtome under RNase-free conditions, and HCR was performed according to the manufacturer’s instructions. Briefly, sections were mounted onto Fisherbrand Tissue Path Superfrost Plus Gold Slides, left to air dry for 3 h, postfixed again in 4% PFA, and dehydrated with a series of ethanol incubations. Sst probes (10 nM) were hybridized overnight at 37 °C in a humidified chamber and then amplified overnight at room temperature. Tissue was imaged on an Olympus BX63 microscope (Olympus Corporation) with a 20× objective and a 5-µm optical section. Images were processed and analyzed in NIH ImageJ software (FIJI). Regions of interest for Sst neurons were manually drawn based on an sfGFP INTACT signal. The average background signal in V1 was calculated for each section and was subtracted from measured Sst mRNA mean gray value (MGV) fluorescence intensity for cells in that section. Wilcoxon rank-sum test was used for statistical analysis. Not significant (ns): *P* > 0.05, *P ≤ 0.05, **P ≤ 0.01, ***P ≤ 0.001, ****P ≤ 0.0001.

## Supplementary Material

Supplementary File

## Data Availability

The snRNA-seq datasets generated in this study are available in the Gene Expression Omnibus (GEO) repository under accession number GSE 196771. This paper does not report original code.
