# Supplementary material for: Single-cell transcriptomic classification of rabies-infected cortical neurons
Source: Proc Natl Acad Sci U S A. 2022 May 24;119(22):e2203677119. doi: 10.1073/pnas.2203677119 (PMC9295789; doi:10.1073/pnas.2203677119)
Supplement: Supplementary File [file pnas.2203677119.sapp.pdf]

Supplementary Information for
Single-cell transcriptomic classification of rabies-infected cortical neurons

Maribel Patiño[1,2,3], Will N. Lagos[1],Neelakshi S. Patne[1], Bosiljka Tasic[4], Hongkui Zeng[4], Edward M. Callaway[1],*

Edward M. Callaway
Email: callaway@salk.edu

This PDF file includes:

Supplementary text
Figures S1 to S2
Legends for supplemental tables

Other supplementary materials for this manuscript include the following:

Table S1 and S2

Supplemental Information

Supplemental tables below are provided separately as Excel files

Table S1. List of Differentially Expressed Genes in Rabies-Infected Nuclei Compared to Control, Related to Figure 1.

Table S2. List of Cell-Type-Specific Differentially Expressed Genes in Rabies-Infected Nuclei Compared to Control, Related to Figure 4.

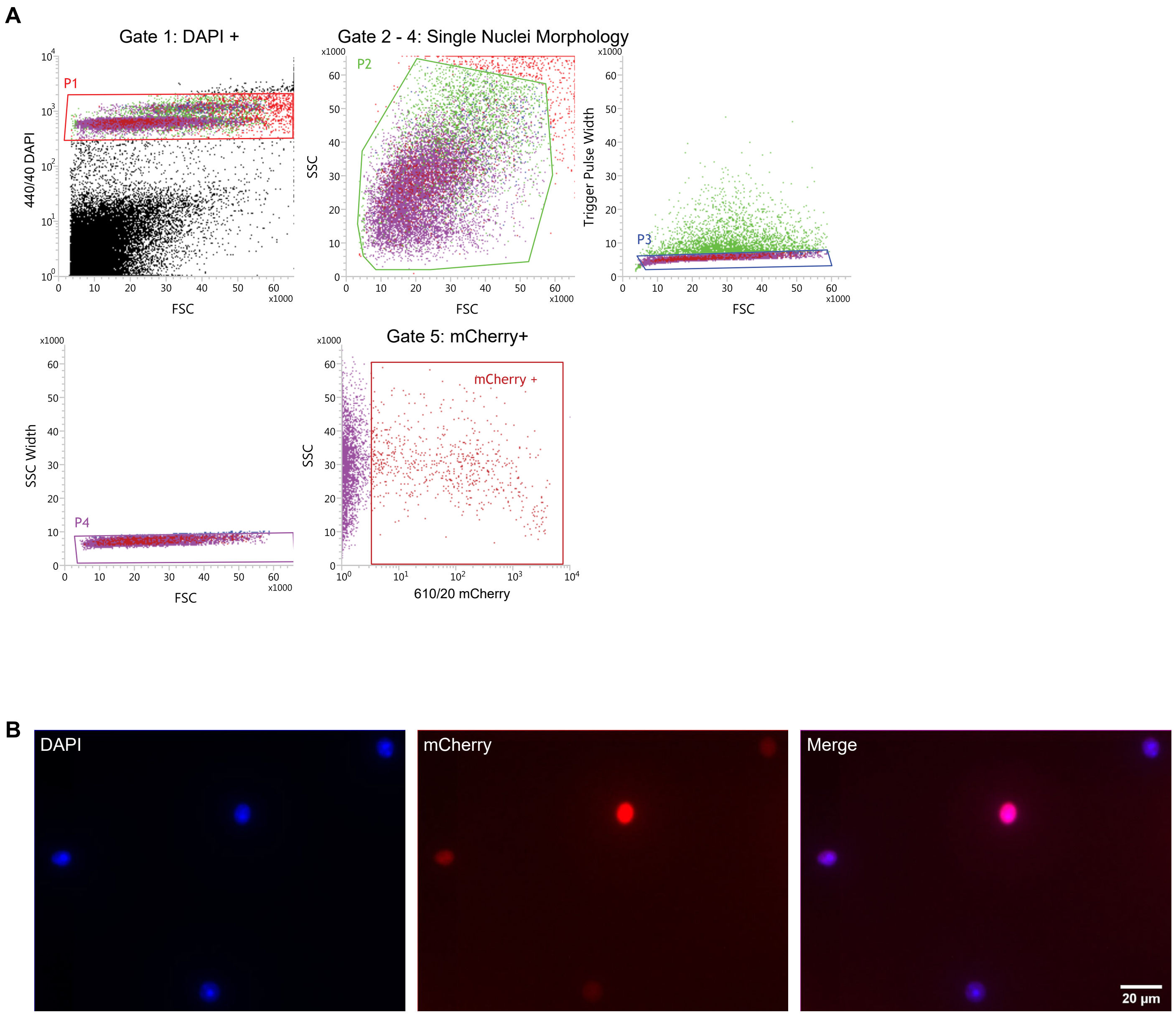

Figure S1. Details on rabies-infected fluorescence-activated nuclei sorting, related to Figure 1

(A) Detailed gating strategy of fluorescence-activated nuclei sorting. Gate 1 selects for DAPI+ nuclei to exclude debris. Gate 2 -4 exclude cell doublets based on single nuclei morphology. Gate 5 selects for mCherry+ fluorescence.

(B) Post-sorting visualization of sorted mCherry+ nuclei for quality control.

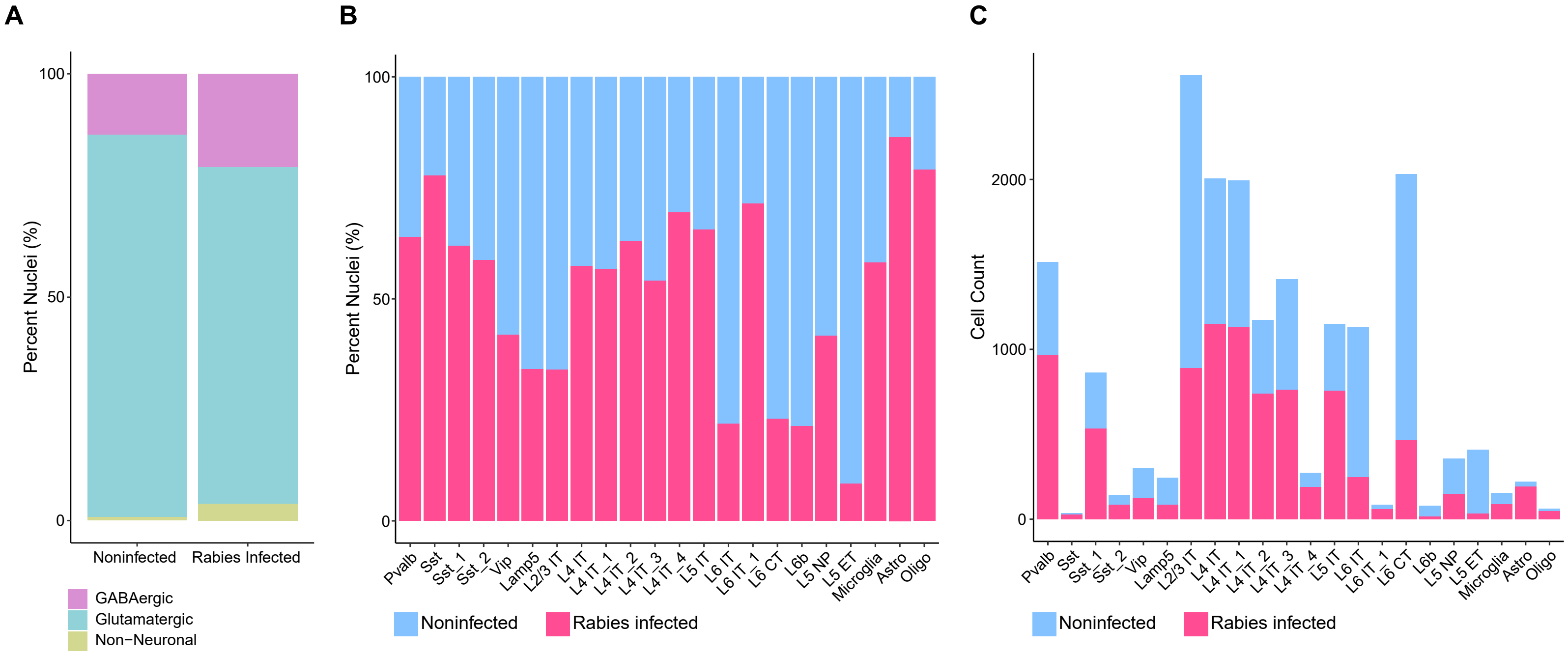

Figure S2. Cell type composition of datasets, related to Figure 2

(A) Percent of excitatory, inhibitory, and non-neuronal nuclei across experimental groups.

(B) Percent distribution of rabies-infected and uninfected nuclei in each cluster.

(C) Absolute count of rabies-infected and uninfected nuclei in each cluster.
